# Optical Concentrators with Simple Layered Designs

**DOI:** 10.1038/srep11015

**Published:** 2015-06-15

**Authors:** Mohammad Mehdi Sadeghi, Lin Xu, Hamid Nadgaran, Huanyang Chen

**Affiliations:** 1 College of Physics, Optoelectronics and Energy & Collaborative Innovation Center of Suzhou Nano Science and Technology, Soochow University, Suzhou, Jiangsu 215006, China; 2 Department of Physics, Shiraz University, Shiraz 71454, Iran

## Abstract

A microwave prototype of field concentrator was recently fabricated, based on the combination of transformation optics and Fabry-Pérot resonances. Perfect electric conductors used as design elements is however, impossible when the working frequencies go to infrared or optical frequencies. Here in this paper, we show that layered structure with alternating dielectrics of positive and negative permittivities can be used to design concentrators of similar function. A practical design with only two kinds of semiconductors is suggested. Theoretical analysis and numerical simulations are performed to verify the concentrating effect.

Optical devices designed by transformation optics^1^,^2^,^3^ have drawn great attention and significant physics interest in recent years. Transformation optics is developed based on invariance of Maxwell’s equations under coordinate transformations^2^ and shows us how to control electromagnetic wave and produce new functionalities by desired permittivity and permeability values. Such devices could be implemented by using highly flexible gradient index metamaterials. Notable examples of these devices are invisibility cloaks^4^,^5^,^6^,^7^,^8^, field rotators^9^,^10^, field concentrators^11^,^12^,^13^,^14^,^15^,^16^ and so on. Among them, invisibility cloak is a topic that has received most scientific attention. Recently the transformation optics theory has also been used to design field concentrators. Theoretical and numerical^11^,^12^,^13^,^14^,^15^ efforts and experimental study^16^ were involved in this topic. In particular, Fabry-Pérot (FP) resonances were used to design and fabricate the first field concentrator, a microwave prototype^16^, where perfect electric conductors (PEC) were used as design elements. However, such a method cannot be extended to design concentrators in higher frequencies, including infrared (IR) or optical frequencies due to the lack of PECs. Here we propose another design for concentrators using layered structure. The required radial anisotropy can be achieved by using alternating dielectric layers of negative and positive permittivities. The advantage is that only two kinds of materials are involved and that layered structure is simple for fabrication with current nanotechnology.

Now we will start from concentrators^11^ using transformation optics and show the idea of concentrators using FP resonances^16^. Then we will introduce layered designs of concentrators. We will solve out the wave equations in every layer analytically and compare the results with numerical simulations from COMSOL Multiphysics. We will study both the lossless case and the loss case of concentrators to show that even for two kinds of dielectrics, the concentrating functionalities are still very good.

## Results

The coordinate transformation for concentrators can be divided into two parts^11^: 1) *r*∈[0,*R*_2_] is compressed into *r*' ∈[0,*R*_1_]; and 2) *r* ∈[*R*_2_,*R*_3_] is extended into *r*' ∈[*R*_1_,*R*_3_]. According to the transformation optics theory, the constitute parameters in *r*' space could be expressed with those in *r* space^2^,


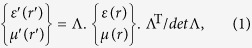


where Λ is the Jacobean transformation matrix. With a linear transformation between *r* space and *r*' space^11^, the parameters of the field concentrator can be written as,


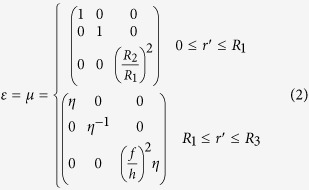


where 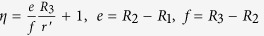 *and h* = *R*_*3*_*−R*_*1*_.

If R_2_**→**R_3_, we shall get *ε*_*r*_**→**∞, and *μ*_*z*_**→**0 for *R*_1_ ≤ *r*′ ≤ *R*_3_^13^,^16^. Such part is called optical void and is not easy to implement^15^,^17^. However, if we only require *ε*_*r*_**→**∞, but allow *ε*_*θ*_ and μ_z_ to be finite values, it should not be difficult to implement thanks to the sound studies on infinitely anisotropic metamaterials^18^,^19^,^20^,^21^,^22^,^23^,^24^. Using such materials to design an optical void, FP conditions should be satisfied along the radial direction, i.e., 
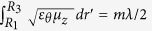
, where *m* is an integer number and λ is the working wavelength, see in details in Ref. [16].

The permittivity or permeability of infinitely anisotropic metamaterials is infinite in one direction and finite in other directions. Let us consider an infinitely anisotropic metamaterial in Cartesian coordinate with a permittivity tensor written as,


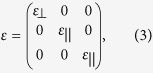


and the case for transverse magnetic (TM) polarization (E_x_, E_y_, H_z_). The wave propagating in the metamaterial could be expressed with *H*_*z*_ *=* *H*_*0*_ *exp(ik*_⊥_*x* + *ik*_||_*y*). The dispersion should be 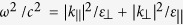.If *ε*_⊥_ goes to infinity, we shall have *ω*^*2*^*/c*^*2*^ *=* |*k*_⊥_|^*2*^*/ε*_||_, which is independent of *k*_||_ . Therefore wave only propagates in the metamaterial in vertical direction (or x direction). When a wave passes through a slab of infinitely anisotropic metamaterials, at FP conditions, there will be a total transmission without any phase change after it leaves the metamaterial. That is why an infinitely anisotropic metamaterial could be regarded as an optical void at FP conditions.

In our case, the concentrator is in a cylindrical shape. The direction coincides with the above vertical direction of infinitely anisotropic metamaterials, which means that wave only propagates along *r* direction in the region of *R*_1_ ≤ *r*′ ≤ *R*_3_.

**Figure 1:**
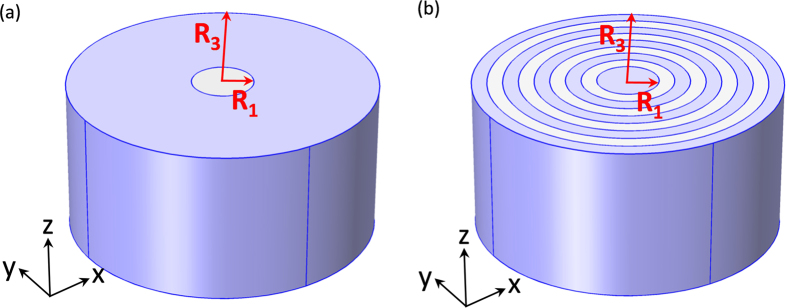
(**a**) Concentrator of effective material parameters. (**b**) Concentrator of layered structure consisting of two kinds of materials A and B with different permittivities.

Now we come to see how to implement such a field concentrator with layered structure (Fig. 1). Suppose we have a concentric layered structure consisting of two kinds of dielectrics A and B with permittivities *ε*_*A*_ and *ε*_*B*_ , respectively, see in Fig. 1b. If the thickness of each layer is thin enough comparing to the wavelength, the structure could be regarded as an effective anisotropic medium^25^,^26^, as denoted in Fig. 1a. From the effective medium theory, we have,


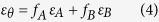



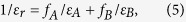


where *f*_*A*_ and *f*_*B*_ are the filling ratios of A and B (*f*_*A*_ +  *f*_*B *_= 1). To get an infinite *ε*_*r*_, 1/*ε*_*r*_ should be zero, which means that *ε*_*A*_ × *ε*_*B *_< 0.

Here we suggest AlInAs and InGaAs as two candidates of materials A and B. Following Ref. [27], we set ε_*A*_ = ε_A1InAs_ = ε_∞−A1InAs_, which is a constant value of 10.23. While 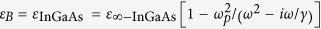, where *ω*_*P*_ is the plasma frequency and *γ* = 0.1 × 10^−12^*s*^−1^ is damping parameter. To ease the design, we first neglect the loss, i.e., let *γ* = 0,


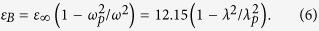


At the plasma frequency (or plasma wavelength *λ*_*P*_ = 8.8 micron), ε_InGasAs_ = 0. To achieve an infinite radial permittivity, ε_B_ should be negative. Therefore we shall set the working wavelength larger than the plasma wavelength. Specifically from equation (5), we have,


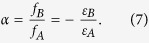


We set a as the relative filing fraction. Therefore *f*_*A*_ = 1/(1 + *α*), *f*_*B*_ = *α*/(1 + *α*), and *ε*_*B*_ = −*αε*_*A*_. Substitute them into equation (4), we get,


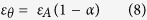


As *ε*_*B*_ = −*αε*_*A*_, by combining equation (6), we have,


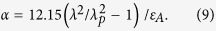


which means that *α* is a function of the wavelength *λ*.

To obtain a field concentrator, the FP conditions should be satisfied,


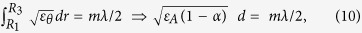


here *μ*_*z*_ = 1. By combining equation (9) and (10), both *α* and the working wavelength could be obtained,


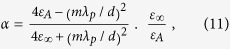



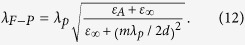


For instance, we set R_1_ = 4 micron and R_3 _= 13 micron (after that ε_*A*_ ≈ (*R*_3_/*R*_1_)^2^, we can simply use AlInAs as the inner core of the concentrator so that the whole system consists of only two kinds of materials). *d* = *R*_3_−*R*_1_ is thereby 9 micron. After that, for each m, we could have an a and *λ*_*F*−*P*_. Table (1) shows *λ*_*F*−*P*_ and related *ε*_*B*_ for each m. When m is smaller than 7, all *ε*_*B*_ are negative and could be use to design concentrators.

**Figure 2:**
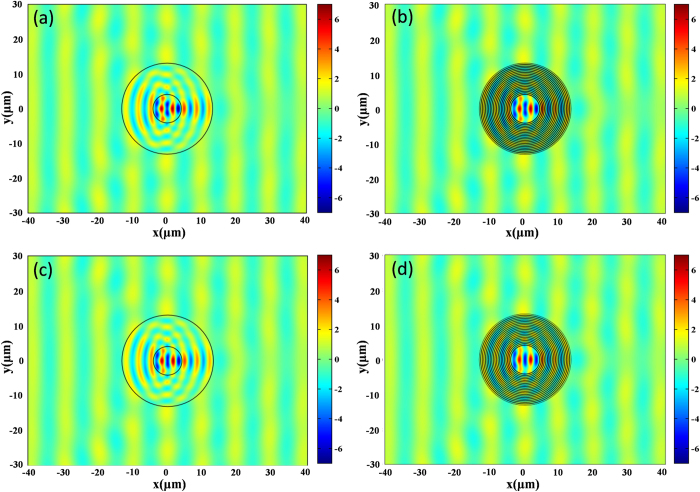
The magnetic field pattern of the concentrator with effective material parameters at (**a**) λ_F−P_ (9.7762 micron) and (**c**) 9.75 micron (based on the first minimum of scattering cross sections), and the magnetic field pattern of the concentrator of the layered structure at (**b**) λ_F−P_ (9.7762 micron) and (**d**) 9.78 micron (based on the first minimum of scattering cross sections). All the materials here are lossless.

As an example, we choose m = 5 in this paper. Therefore, *α* = 0.278 and *ε*_*θ*_ = 7.386. Firstly, as shown in Fig. 2a, we plot the magnetic field pattern for an incident TM plane wave interacting with a concentrator of an effective anisotropic medium with *ε*_*r*_ = 10000 and *ε*_*θ*_ = 7.386. We found that the field is enhanced in the core medium (with *ε*_*A*_ = 10.23) while the whole structure is almost invisible. To realize such a concentrator, as we have discussed before, only two kinds of alternating semiconductors AlInAs and InGaAs are required. In Fig. 2b, we plot the *H*_*Z*_ field pattern for such a layered structure, where 20 unit cells are used. From the pattern, we see that the layered design perform a good concentrating effect as that with effective parameters. Both the working wavelengths in (a) and (b) are the calculated *λ*_*F*−*P*_ (9.7762 micron).

**Figure 3:**
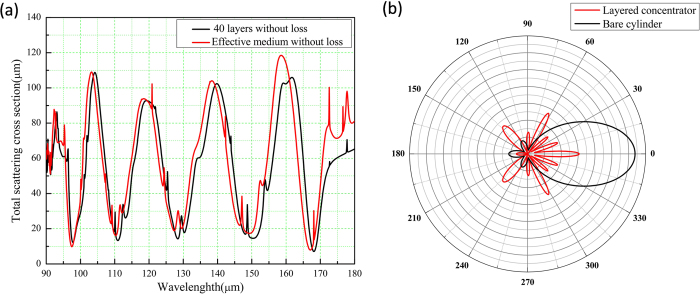
(**a**) Total scattering cross sections of concentrators for both layered structure (black curve) and effective medium (red curve) at different wavelengths. (**b**) Angular scattering cross sections of layered concentrator (red curve) and the core medium (black curve) at 9.78 micron. All the materials here are lossless.

Furthermore we solve out the wave equation for the above layered structure. The scattered magnetic field should be written as,


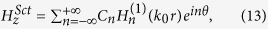


where *C*_*n*_ is the coefficient we sort out after matching the boundary conditions at the interfaces of each layer, and 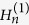 is the n-th order Hankle functions of the first kind. The far-field scattering cross section is defined as,


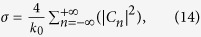


which was plotted for different wavelengths (the black curve in Fig. 3a). The first minimum is at the wavelength of 9.78 micron, which is consistent to the theoretical calculated *λ*_*F*−*P*_. We also plot the magnetic field pattern at this wavelength, as shown in Fig. 2d, where the concentrating effect is clearly seen as well. To make a comparison, we further plot the scattering cross section for different wavelengths for the effective medium, as shown by the red curve in Fig. 3a. The first minimum is now at the wavelength of 9.75 micron. The related magnetic field pattern at this wavelength is plotted in Fig. 2c. Other minima of both curves in Fig. 3a are corresponding to other orders of FP resonances (from m = 1 to 4). Therefore, we see that the designed concentrator works for multi-wavelengths as the original design using PECs^16^.

To show the functionality more clearly, we also calculate the angular scattering cross section of the layered design at the first minimum,


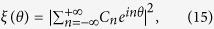


and plot it in Fig. 3b (the red curve). For most directions, the scattering cross section is smaller than that of a bare core medium (denoted by the black curve in Fig. 3b).

**Figure 4:**
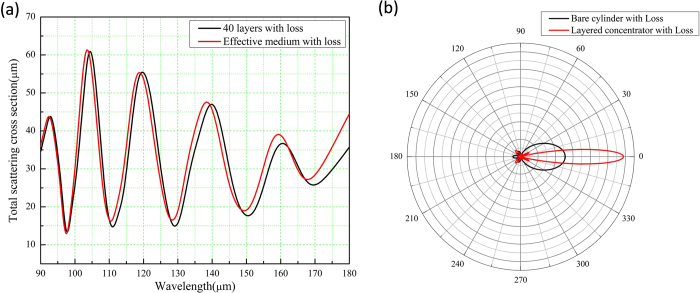
(**a**) Total scattering cross sections of concentrators for both layered structure (black curve) and effective medium (red curve) at different wavelengths. (**b**) Angular scattering cross sections of layered concentrator (red curve) and the core medium (black curve) at 9.78 micron. The concentrator here consists of lossy materials.

Now, we come back to common materials and consider the effect of loss. In Fig. 4a, we plot the total scattering cross sections of the concentrators for both the layered structure and the effective anisotropic medium at different wavelengths. The black curve is for the case of layered structure, while the red curve is for that of effective anisotropic medium. The first minimum of both cases is at about 9.78 micron. We therefore choose this wavelength and plot the angular scattering cross section of the layered concentrator as shown by the red curve in Fig. 4b. For comparison, we also plot that of a bare core medium, as denoted by the black curve, Again, we find that for most directions, the concentrator can reduce the scattering of the core medium. However, for the forward direction, the scattering is enhanced, that is due to the loss of the concentrator.

**Figure 5:**
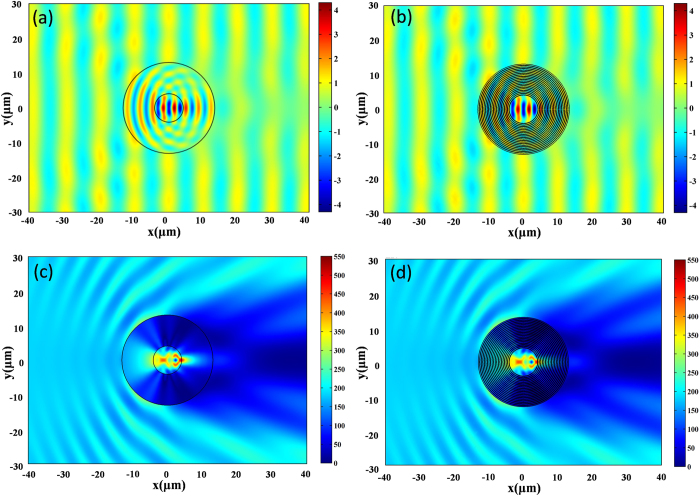
The magnetic field pattern of the concentrator with (**a**) effective material parameters and (**b**) layered structure at 9.78 micron (based on the first minimum of scattering cross sections), and the power flow distribution of the concentrator with (**c**) effective material parameters and (**d**) layered structure at 9.78 micron. The concentrator here consists of lossy materials.

We plot the *H*_*Z*_ field patterns of the concentrators for both the effective anisotropic medium and the layered structure for the first minimum in Fig. 5a,b, respectively. From the figures, we find that although there is a shadow in the forward direction for both cases due to the loss, the field is still enhanced in the core medium. To demonstrate it more clearly, we also plot the power flow distributions for both the effective medium and the layered design in Fig. 5c,d, respectively. Therefore the current design can more or less function as a practical concentrator.

## Discussion

In summary, we have proposed a layered design for field concentrators by using only two kinds of semiconductors. Such concentrators function well for multi-wavelength. The concentrating effect is verified by both theoretical analysis and numerical simulations. The design is simple. We expect that there will be a real construction in the coming future.

## Additional Information

**How to cite this article**: Sadeghi, M. M. *et al*. Optical Concentrators with Simple Layered Designs. *Sci. Rep*. **5**, 11015; doi: 10.1038/srep11015 (2015).

## Figures and Tables

**Table 1:** Parameters for each m.

*m*	*λ*_*F−P*_	*ε*_*B*_
1	1.1827e-05	−9.7976
2	1.15e-05	−8.5953
3	1.1007e-05	−6.8596
4	1.0414e-05	−4.8665
5	9.7762e-06	−2.8450
6	9.1354e-06	−0.9439
7	−0.0745	+0.7624
